# Supplementary material for: Time-Lapse Flow Cytometry: A Robust Tool to Assess Physiological Parameters Related to the Fertilizing Capability of Human Sperm
Source: Int J Mol Sci. 2020 Dec 24;22(1):93. doi: 10.3390/ijms22010093 (PMC7796328; doi:10.3390/ijms22010093)
Supplement: Supplementary file 1 [file ijms-22-00093-s001.pdf]

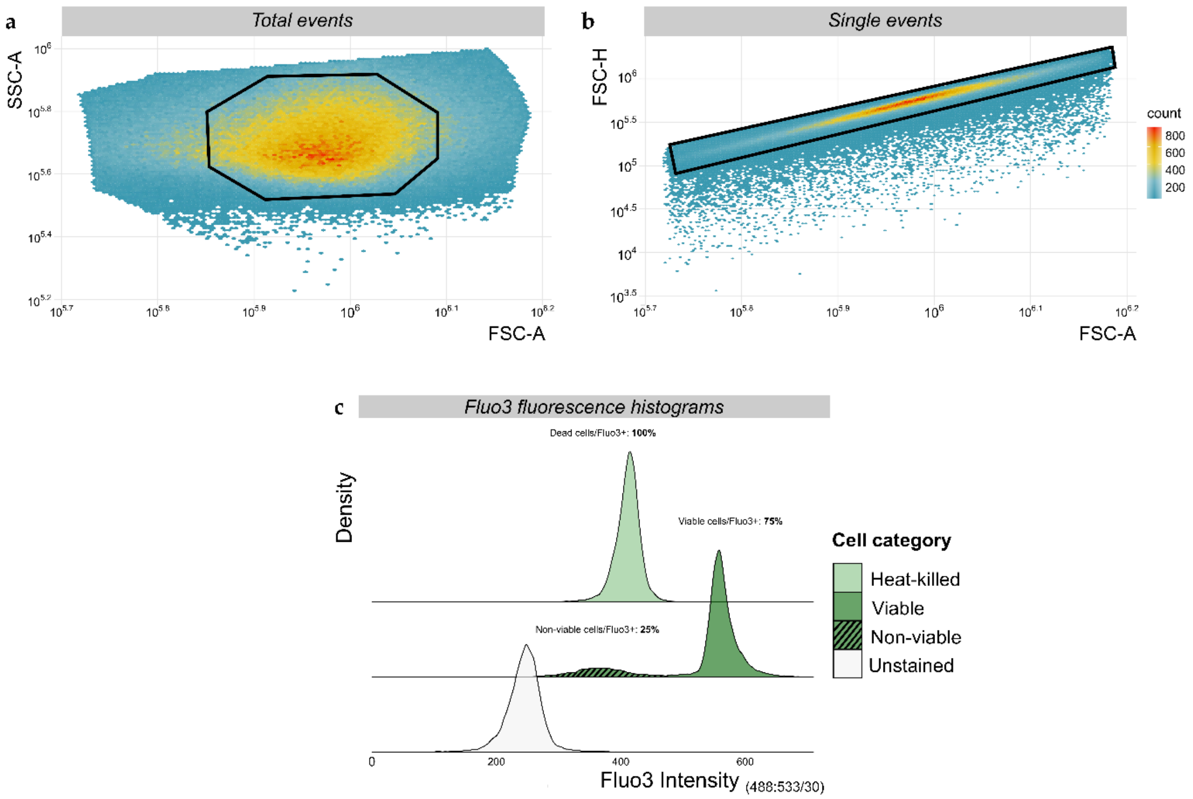

Figure S1. Data acquisition and selection of viable single human sperm cells in the BD Accuri C6 Plus flow cytometer. (**a**) Representative density plot comparing side (SSC-A) versus forward scatter area (FSC-A). Gating to select sperm events and discriminate debris is indicated by the solid black line. (**b**) Forward scatter height (FCS-H) versus FSC-A profile applied to the subpopulation selected in **a** to exclude aggregates and select single events. (**c**) Density histograms of Fluo3-AM (Fluo3) fluorescence emission for cell samples that were unstained (white), or Fluo3-stained and either untreated (dark green) or heat-killed (light green). The percentage of viable, non-viable or dead cells is indicated above the histograms when appropriate.
